# Supplementary material for: Poliomyelitis in Nigeria: Impact of Vaccination Services and Polio Intervention and Eradication Efforts
Source: Vaccines (Basel). 2025 Feb 25;13(3):232. doi: 10.3390/vaccines13030232 (PMC11945573; doi:10.3390/vaccines13030232)
Supplement: Supplementary file 1 [file vaccines-13-00232-s001.zip › vaccines-3440521-supplementary.pdf]

Supplementary Materials:

Table S1. Literature Search Methodology Using PICOS Framework.

PICOS FRAMEWORK

FEATURES	KEY SEARCH TERMS
POPULATION	Nigeria
INTERVENTION	Polio Vaccination Services, Polio Intervention Policies
COMPARATOR	Not Relevant to Search Strategy as Study is an Exploratory and Single-Intervention Study
OUTCOME	Vaccine Hesitancy, Vaccine Uptake, Polio Eradication, Utilization, Impact, Challenges, Poliomyelitis, Polio Outbreak
STUDY DESIGN	Any as Research Question is Broad and Exploratory

Table S2. Search Strategies For Ovid Medline Literatures (Ovid MEDLINE(R) and Epub Ahead of Print, In-Process, In-Data-Review & Other Non-Indexed Citations, Daily and Versions 1946 to April 09, 2024).

#	Query	Resultsf from 9 Apr 2024
1	exp Nigeria/	35,100
2	"Nigeria*".ab,kw,ti.	45,215
3	"Nigeria* popula*".ab,kw,ti.	661
4	1 or 2 or 3	50,420
5	Vaccination/ or Immunization Programs/ or PoliovirusVaccines/ or Immunization/	166,709
6	Communicable Disease Control/ or PopulationSurveillance/ or Primary Prevention/	114,019

7	(polio* adj3 vaccin*).ab,kw,ti.	7,327
8	(Polio* adj3 program*).ab,kw,ti.	553
9	"Polio* eradicat* initiat*".ab,kw,ti.	527
10	(Polio* adj3 prevent*).ab,kw,ti.	362
11	(polio* adj5 effort*).ab,kw,ti.	292
12	"intervent* polic*".ab,kw,ti.	765
13	(polio* adj5 polic*).ab,kw,ti.	100
14	(polio* adj5 serv*).ab,kw,ti.	88
15	5 or 6 or 7 or 8 or 9 or 10 or 11 or 12 or 13 or 14	279,795
16	4 and 15	1,898
17	Vaccination Refusal/ or Vaccination Hesitancy/	1,866
18	Vaccination Coverage/	2,523
19	Disease Eradication/	3,559
20	Polio/ or Poliovirus/	25,976
21	"Polio* eradicat$3".ab,kw,ti.	1,974
22	"Vaccin* uptake".ab,kw,ti.	6,874
23	(Eradicat* adj3 polio*).ab,kw,ti.	2,750
24	(Polio* adj3 effect*).ab,kw,ti.	549
25	"Polio* free".ab,kw,ti.	437
26	(Inciden* adj3 polio*).ab,kw,ti.	381
27	"Polio* outbreak".ab,kw,ti.	229
28	(Polio* adj5 challenges).ab,kw,ti.	139
29	(Prevalen* adj3 polio*).ab,kw,ti.	127
30	(impact* adj5 polio*).ab,kw,ti.	125
31	(Effect* adj3 polio* vaccin*).ab,kw,ti.	111
32	"Polio* endemic*".ab,kw,ti.	120
33	(Polio* adj3 impact).ab,kw,ti.	57
34	17 or 18 or 19 or 20 or 21 or 22 or 23 or 24 or 25 or 26 or 27 or 28 or 29 or 30 or 31 or 32 or 33	40,221
35	16 and 34	571
36	limit 35 to (english language and yr="2018 - 2024")	197
